# MiRNA-221 from tissue may predict the prognosis of patients with osteosarcoma

**DOI:** 10.1097/MD.0000000000011100

**Published:** 2018-07-20

**Authors:** Ningji Gong, Mingzhi Gong

**Affiliations:** Department of Emergency, The Second Hospital of Shandong University, Jinan, Shandong, China.

**Keywords:** miR-221, osteosarcoma, prognosis

## Abstract

This study was aimed to investigate the relationship between miR-221 expression and prognosis in patients with osteosarcoma.

miR-221 expression in 69 osteosarcoma specimens and corresponding noncancer tissues were characterized by quantitative reverse transcription polymerase chain reaction. The associations of miR-221 expression with clinicopathologic factors and prognosis in patients with osteosarcoma were statistically analyzed.

miR-221 expression in patients with osteosarcoma was significantly higher than in the corresponding noncancer tissues (*P* < .01). miR-221 overexpression was significantly associated with tumor stage, metastatic status, and response to chemotherapy pretreatment. Cox regression analysis revealed that miR-221expression, metastasis, and response to chemotherapy were independent prognostic indicators for osteosarcoma.

miR-221 upregulation may predict clinical outcomes in patients with osteosarcoma.

## 1 Introduction

Osteosarcoma mainly arises from the metaphysis of the long bones and it is the most common primary bone malignancy in adolescents and young adults.^[1,2]^ Despite advances in disease management and treatment, the long-term prognosis of patients with osteosarcoma is still poor. Approximately 80% of patients eventually develop recurrent metastatic osteosarcoma following surgical treatment^[3]^ and the 5-year survival rate of these patients is only 50% to 60%.^[4]^ Combined neoadjuvant chemotherapy has greatly improved the survival rate. However, the prognosis of patients with a poor response to chemotherapy is still unfavorble.^[5]^ Although several molecular targeted drugs have emerged, none of them have been well established for the treatment of osteosarcoma. Moreover, the molecular events characterizing the development and propagation of osteosarcoma remain obscure.^[6]^ Therefore, there is an urgent need to further explore the molecular mechanisms involved in the development of osteosarcoma, its recurrence, and metastasis to improve therapies.

MicroRNAs (miRNAs) are small noncoding RNAs of 18 to 25 nucleotides in length, transcribed from nonprotein coding genes or introns, which regulate gene expression at a posttranscriptional level by binding to the 3′-untranslated regions of their target mRNAs.^[7]^ It has been reported that miRNAs are potential regulators of a wide range of biologic processes including development, cell differentiation, proliferation, and apoptosis.^[8–14]^ To date, several human miRNAs such as miR-335, miR-145, and miR-128 have been shown to be dysregulated in osteosarcoma,^[15–17]^ contributing to the development and progression of osteosarcoma. MiR-221 is encoded in tandem from a gene cluster located in the chromosome Xp11.3.^[18]^ Accumulating evidence shows that miR-221 may play an important role in human tumorigenesis. Its upregulation has been reported in various malignancies, including glioblastoma, cutaneous melanoma, lymphoma, papillary thyroid carcinoma, and breast cancer.^[19–25]^ In addition, high miR-221 expression is correlated with poor prognosis in glioma, breast cancer, hepatocellular carcinoma and pancreatic cancer.^[26–32]^ However, the role of miR-221 in the prognosis of osteosarcoma has not been fully clarified. Therefore, the aim of this study was to investigate the clinicopathologic and prognostic value of miR-221 in human osteosarcoma.

## 2 Materials and methods

### 2.1 Patients and tissue specimens

The present study was approved by the Research Ethics Committee of The Second Hospital of Shandong University. A total of 69 paraffin-embedded osteosarcoma specimens and corresponding noncancer tissues were collected from patients at the Department of Orthopedic Surgery, the Second Hospital of Shandong University, China, from January 2005 to January 2012. All specimens remained anonymous according to the ethical and legal standards. All the tissue samples were reevaluated according to the WHO classifications by 2 pathologists. In the follow-up period, overall survival was measured from diagnosis to death or last follow-up. Clinical information, such as age, gender, location, pathology, and response to chemotherapy, was also collected. Clinical follow-up was available for all patients (median, 19 months; range, 7–50 months). At the end of the follow-up, 37 patients were still alive, while 32 died. Overall survival was calculated from the date of the initial surgical operation to death. All patients who died from other diseases or unexpected events were excluded from this study.

### 2.2 miRNA quantitative reverse transcription polymerase chain reaction

miR-221 expression in the osteosarcoma and corresponding noncancer tissues was determined by quantitative reverse transcription polymerase chain reaction (qRT-PCR). Total RNA was extracted from paraffin-embedded samples using the acid phenol-chloroform method. In brief, total RNA was extracted using of TRIzol (Invitrogen, Carlsbad, CA) and digestion buffer (30 mmol/L tris(hydroxymethyl)aminomethane-HCl [pH 7.5], 20 mmol/L EDTA [pH 8], and 1% sodium dodecyl sulfate in 0.1% diethyl pyrocarbonate-treated water) according to the supplier's instructions. miR-221- and RNU6B (as internal control)-specific cDNA were obtained from total RNA using gene-specific primers according to the TaqMan MicroRNA assays protocol (Applied Biosystems, Foster City, CA). The reverse transcription products were then amplified and detected by qRT-PCR using Taqman MicroRNA assay (Applied Biosystems) specific for hsamiR-221. Relative mRNA expression was calculated by the 2^-△△Ct^ method using an Applied Biosystems 7500 real-time PCR system (Applied Biosystems). The median value of the relative miR-221 expression was used as a cutoff point to divide the 69 osteosarcoma patients into low and high expression groups.

### 2.3 Statistical analysis

All statistical analyses were performed using SPSS software version 18.0 (SPSS Inc, Chicago, IL). The correlation between miR-221 and clinical-pathologic indexes was assessed using Spearman rank tests. Survival curves were plotted using the Kaplan–Meier method, and differences between survival curves were tested using the log-rank test. Cox proportional hazards model was used to identify the factors that have significantly independent influence on survival. *P* < .05 was considered statistically significant.

## 3 Results

In the present study, we investigated miRNA-221 dysregulation in surgically resected and paraffin-embedded osteosarcoma samples and analyzed their expression levels in the context of the potential as clinically relevant biomarkers.

**Figure 1:**
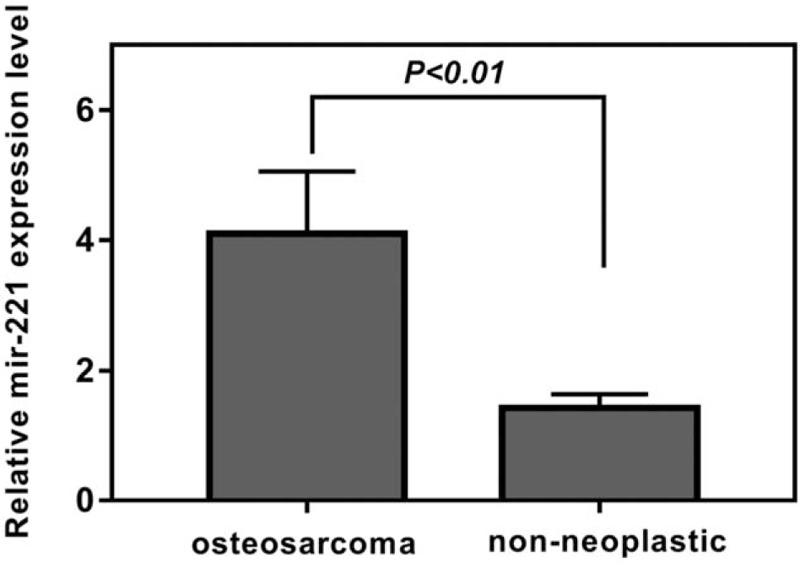
Relative miR-221 expression in human osteosarcomas (n = 69) and adjacent normal tissues (n = 69). All tumor specimens were obtained from the center of the tumor. The expression of miR-221 was normalized to that of the U6B small nuclear RNA gene (*RNU6B*) control.

miR-221 expression in osteosarcoma and adjacent healthy tissues was analyzed by qRT-PCR. miR-221 exhibited a relative high expression in osteosarcoma than in healthy tissue (*P* < .01; Fig. 1). Median mir-221 value was used to divide patients with high and low mir-221 expression.

Relationships between miR-221 expression and various clinicopathologic features in osteosarcoma are shown in Table 1. A total of 69 patients with osteosarcoma with a median age of 21 years were included in the study. miR-221 expression increased with increasing tumor stage. In addition, its expression was significantly higher in patients with stages IIB/III tumor than stage IIA. miR-221 overexpression was significantly associated with metastatic status and response to chemotherapy pretreatment. However, no significant association was found between miR-221 levels and pathologic type, age, gender, or location.

**Table 1:**
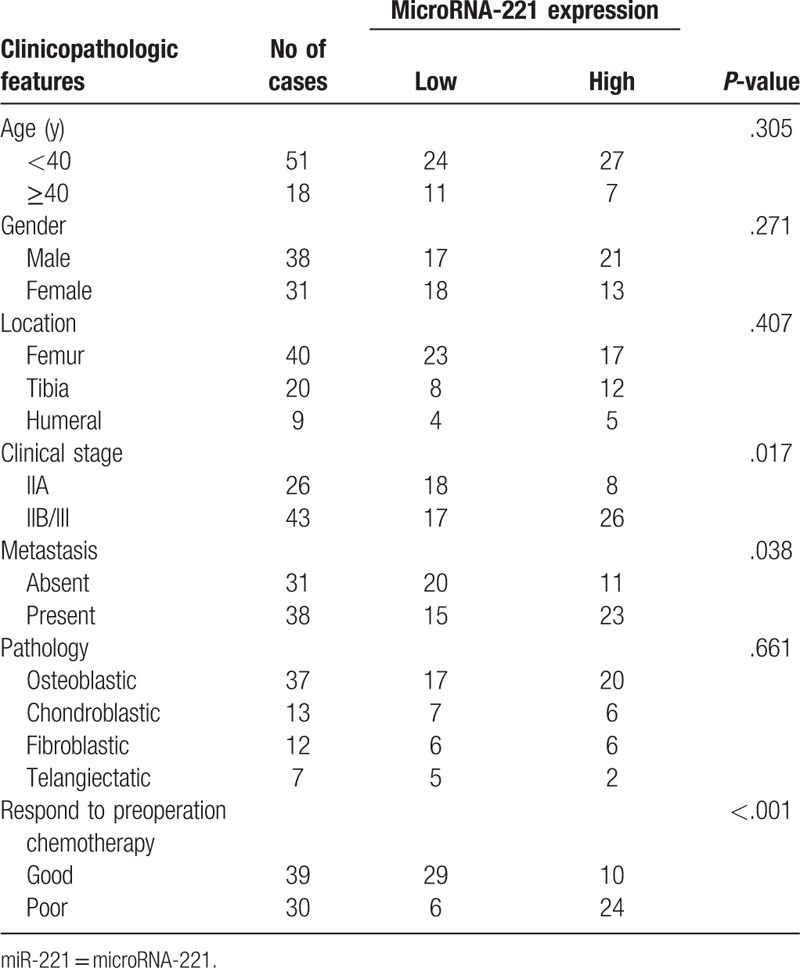
Relationship between miR-221 expression with clinicopathologic features.

**Figure 2:**
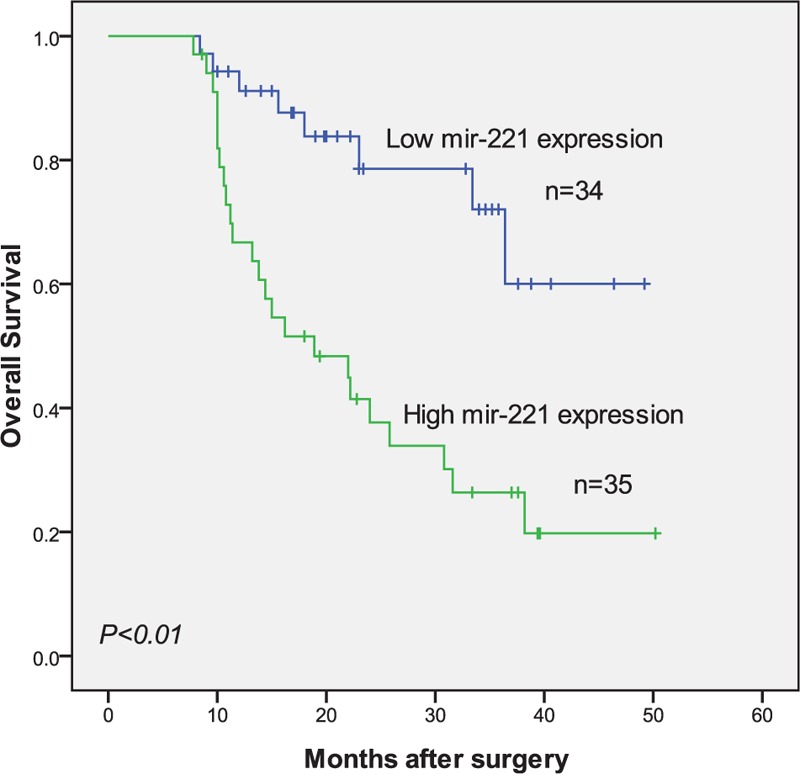
Kaplan–Meier curves for overall survival in patients with osteosarcoma divided according to miR-221 expression.

Association of high miR-221 expression with prognosis in patients with osteosarcoma was also analyzed. During the follow-up, the clinical information of patients with miR-221 high or miR-221 low osteosarcoma was analyzed. At the end of the study period, 37 of the 69 patients were still alive, while 32 patients died during the study period. Kaplan–Meier curve shows that the overall survival (*P* < .01; Fig. 2) of patients with high miR-221 expression was lower than those with low miR-221 expression.

**Figure 3:**
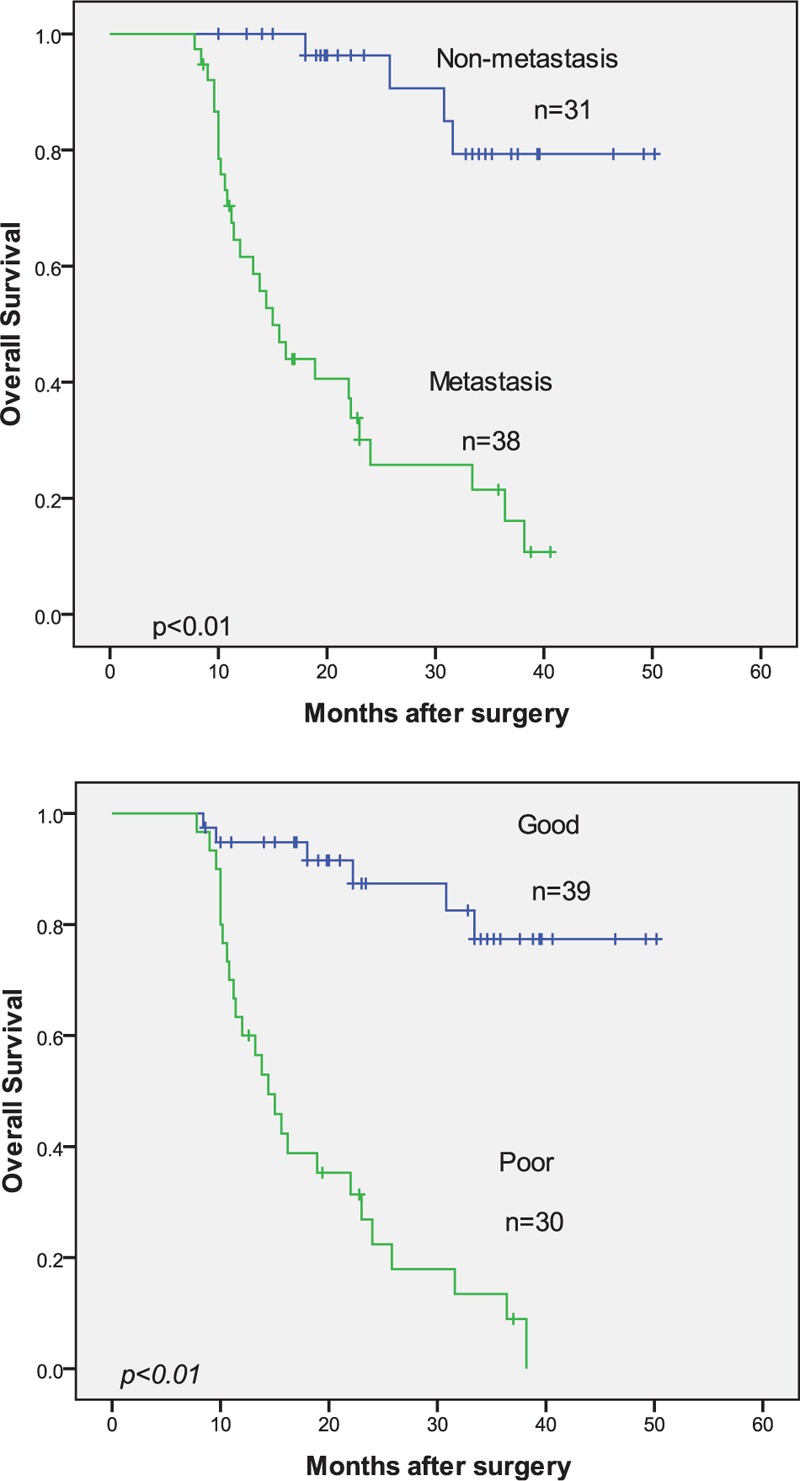
Kaplan–Meier curves for overall survival in patients with osteosarcoma divided according to metastasis and response to chemotherapy.

In addition, metastasis and response to chemotherapy were also found to be associated with the overall survival of patients with osteosarcoma (*P* < .01; Fig. 3). Moreover, Cox regression analysis was performed to determine the association of miR-221 expression and clinical-pathologic index with the overall survival of patients with osteosarcoma. Both univariate and multivariate analyses revealed that miR-221 expression, metastasis, and response to chemotherapy were independent prognostic indicators for osteosarcoma (Table 2).

**Table 2:**
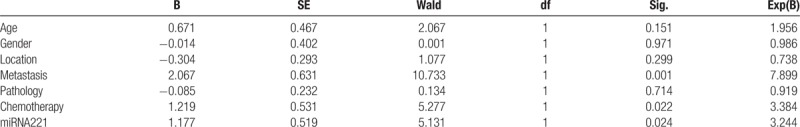
Univariate and multivariate analysis of prognostic parameters in patients with osteosarcoma by Cox regression analysis.

## 4 Discussion

Osteosarcoma is a severe bone cancer characterized by a poor clinical outcome. Therefore, there is an urgent need to explore the molecular mechanisms involved in the development of osteosarcoma to improve therapies. A lot of studies showed that high miR-221 expression was correlated with tumor size, stage, and overall survival. High miR-221 expression is related to a trend of invasion, earlier metastasis, and shorter time to recurrence.^[23,27,33–37]^ Guo's study reported a reverse correlation between circulating miR-221 level and survival after treatment for patients with natural killer/T-cell lymphoma.^[21]^ Other study found that circulating miR-221 levels were overexpressed in patients with malignant melanoma. Moreover, there was a decrease of miR-221 levels after surgical removal of the primary tumor, and increase again at recurrence.^[38]^ In our study, we examined miR-221 expression in patients with osteosarcoma and found that miR-221 was upregulated in osteosarcoma tissues other than adjacent healthy tissues, and in patients with advanced stages and metastasis, which is consistent with several published studies. These findings indicated that miR-221 might play some roles in the invasion of osteosarcoma cells.

Similar with other miRNAs, the mechanisms behind altered miR-221 expression in cancer may be the regulation of several protein coding genes. The molecular mechanisms that link miR-221 overexpression to tumorigenesis and development are not well understood. Several functional targets have been validated. Recent studies indicated that miRNA-221 overexpression promotes cancer cell proliferation by suppressing the expression of the cyclin-dependent kinase inhibitors CDKN1B/p27 and CDKN1C/p57, which are important regulators of cell cycle progression.^[39,40]^ MiR-221 can also promote cell-cycle progression in cancer cells and facilitates cell proliferation via downregulation of p27Kip1 and p57Kip2, cell-cycle inhibitors, and tumor suppressors.^[39,41]^ In addition, Yoon et al demonstrated that miR-221 directly inhibits the posttranscriptional expression of metallopeptidase inhibitor 3, an inhibitor of matrix metalloproteinases, and plays an important role in promoting the invasion of human gliomas.^[36]^ Furthermore, miR-221 promotes breast cancer cell proliferation and invasion and suppresses cell apoptosis by regulating its target gene ARHI.^[42]^ The oncogenic effect of miR-221 is also mediated by phosphatase and tensin homolog (PTEN).^[38]^ Moreover, miR-221 upregulation induces cell survival and cisplatin resistance through PI3K/Akt pathway in human osteosarcoma, suggesting a potential target for the treatment of this malignancy.^[43]^ Some reports also demonstrated the association between miR-221 and tamoxifen resistance in breast cancer.^[44]^

Deregulation of miRNA expression has been shown in different human cancer; however, the molecular mechanism underlying regulation of miRNA expression in cancer is not completely understood. Studies showed miRNAs may be regulated by epigenetic mechanism including abnormal methylation and histone modifications, gene mutations involved in the processing, and maturation of microRNAs, or regulation of miRNA stability.^[45–47]^

Eva Bandres et al have first demonstrated that suppression of hsa-miR-9, hsa-miR-129, and hsa-miR-137 genes in colorectal cancer is mediated in part by epigenetic mechanisms, including DNA hypermethylation and histone acetylation.^[48]^ MiR-221 is clustered on the X chromosome and it has been reported to be overexpressed in many cancers; however, as far as we known, studies often focused on elucidating the function of miR-221 in human osteosarcoma, there is rare mechanism data illustrating the detailed reason for the dysregulated expression of miR-221 in osteosarcoma. Exploratory researches uncovering the molecular mechanism underlying the regulation of miR-221 expression may offer important information in the diagnosis and treatment of osteosarcoma.

The progression of osteosarcoma is a complicated process, but miR-221 might serve as an important role in this intricate network. In conclusion, the present study showed that miR-221 expression was increased in osteosarcoma samples, and miR-221 overexpression was associated with clinical stage and metastasis. Furthermore, higher miR-221 level was associated with a less favorable prognosis. These findings indicated that miR-221 might serve as a novel diagnostic and prognostic marker, or therapeutic target in osteosarcoma.

## Author contributions

**Data curation:** Ningji Gong.

**Funding acquisition:** Mingzhi Gong.

**Investigation:** Ningji Gong.

**Resources:** Mingzhi Gong.

**Software:** Ningji Gong.

**Validation:** Mingzhi Gong.

**Writing – original draft:** Ningji Gong.

**Writing – review & editing:** Mingzhi Gong.
